# Round the Hospitals

**Published:** 1920-06-26

**Authors:** 


### ROUND THE HOSPITALS.

A large meeting gathered at the hall of the Royal Society of Medicine, June 17, to receive Sir Arthur Stanley's congratulations on the successful operations of the College of Nursing. It was a memorable meeting because it was the first at which every member of the Council of the College had come to be elected by the votes of the members. The only note of regret was over the voluntary retirement of the prime originator of the College, Dame Sarah Swift, who refused nomination on the ground that new blood is required, but everyone hopes the College may not lack the inspiration of her presence on the Council for more than one year. That Dame Sarah was marked out by genius and

**ROUND THE HOSPITALS**—(*continued*).

experience, as well as by those indefinable signs which indicate greatness of character; to do much for her profession was apparent years ago to such as knew her work at Guy's. It must have been a little disconcerting to such as persistently treat the chairmen of our voluntary hospitals as the enemies of nurses to find Lord Knutsford elevated to the Council by ballot taken among 17,000 nurses. The election of a Queen's nurse as well as a school nurse will be welcomed by many who desired to see these branches of work represented. Another Queen's nurse was elected for Ireland, and a much-needed ward sister for Glasgow, and the results of the ballot were, generally speaking, highly popular.

---

The College Conferences, held after the annual meeting and the following morning, were exceedingly well attended, and those present were thoroughly alive and alert. Representatives of the nursing profession from all over the country were present. There was hardly time for anything in the nature of general discussion, but many facets of the selected subjects were dealt with and divergent views freely expressed. The evening session, devoted to the best methods of drawing women into the profession, was of exceptional interest. Dr. Janet Lane-Claypon's address and her method of delivery were excellent, and her finished method and wide view of the whole subject made many rejoice to know that she is so closely associated with the work at King's College for Women of moulding nurses to be sister-tutors. Miss Musson spoke temperately and wisely of the improvements which can be effected in the training-schools with a view to making probationership more attractive. Miss Edmondson astonished many of her hearers by insisting that all kinds of nursing —children, fever, mental and midwifery—should be embraced in the one training, and that all the housemaid's work should be cut out of the nurse's training. She stated, moreover, that in Aberdeen this reform had been brought about and that the nurses enjoyed one day's rest in seven.

---

The Conference on June 18 on District Nursing brought out some very interesting facts, chief among which was Miss Hancox's statement that there has recently been a rapid development of district nursing and that a very different type of woman has been induced to take up the work since it has become so closely interwoven with civic hygiene. Miss G. R. Skinner, who pleaded, as did Miss Grace Vaughan, for smaller districts and better modes of transit for district nurses, paid a deserved tribute to the devotion of village nurse-midwives. Miss Tait McKay, R.R.C., urged the importance of courage among "the people's guides" and a large outlook. More than one speaker urged that hospital matrons should be more sympathetic in the matter of district nursing, both as regards facilities for training and encouragement to the newly-certificated to join this branch of the public service.

Every member of the nursing services will learn with intense gratification that the most honourable of all decorations, that of the Victoria Cross, is now open to women who distinguish themselves by valour in the face of the enemy. The King has graciously decided that for the future, under conditions analogous to those which govern the bestowal of the V.C. on men, it shall be open to nurses of the Imperial Naval or Military Services, or any woman attached in an officially recognised capacity to the sea or land or air forces. The Victoria Cross was instituted in 1856. It consists of a bronze maltese cross, with Royal crest (lion and crown) in centre, and below it a scroll inscribed For Valour. The conspicuous bravery which constitutes a claim to it must be exhibited "by some signal act of valour or devotion" to the country "performed in the presence of the enemy." In the case of recipients who are not of commissioned rank the Cross carries with it a pension of £10 a year, with an additional £5 a year for each clasp; larger grants are sometimes given to recipients who are in need.

---

The General Nursing Council for Scotland has got already so far as to issue its draft rules for the conduct of the business of the Council, an abstract of which will be found on page 332. They are of a purely formal character for facilitating despatch of business and guarding against any irregularity in its conduct. The Council have power to appoint such committees as they think fit, and the detailed work of the Council will in natural course be delegated to these committees, on which, with the exception of the Finance Committee, the Council has power to appoint "other persons with special knowledge," not members of the Council. This will afford an opportunity for securing the co-operation of some whose names were regretfully omitted from the Council, and should materially broaden the basis of deliberation on the many important problems awaiting solution.

---

Everyone will be glad to hear that the financial difficulties which stood in the way of securing the proposed V.A.D. Club have been overcome. The premises at 28 Cavendish Square, containing thirty-five to forty bedrooms, were secured, after all, owing to the energy and resource displayed by Lady Ampthill and her co-directors, and the Club was opened on June 15. A distinguished company of over 100 guests assembled at luncheon, at which Lady Ampthill presided, and she was able to announce that the Queen has consented to be Patroness of the Club, while Princess Mary, who is Commandant of a Detachment, has become an honorary member. The membership already exceeds 2,500. Many general service V.A.D.s are among the staff, and unexpected recognitions among members who have seen service in almost every part of the world are the order of the day.

---

Extraordinary interest was shown in Plymouth in the proceedings of the Town Council on June 14 relating to the outbreak of puerperal fever at the

**ROUND THE HOSPITALS**—*(continued)*.

St. James's Terrace Maternity Home, conducted by the Three Towns Nursing Association. Three cases, all of which terminated fatally, occurred at the Home in November and December last year, and a further case occurred in May this year, but was removed to the Borough Isolation Hospital and recovered. An inquiry into the circumstances has recently been held in response to a request for a detailed report from the Ministry of Health, and the Town Council had under consideration the report furnished by the committee appointed for this purpose. The committee censure the managers for admitting a septic case of mammary abscess to the home, and also for the lack of precaution shown in not suspending the midwife for a longer period after the first case and for continuing to take patients after the second death. They further censure the medical officer of health for delay in visiting the home and for failure to deal promptly with the matter. An amendment disapproving of the clause relating to the medical officer of health was carried by twenty-two votes to twenty. Several of the speakers spoke in high terms of the Three Towns Nursing Association, and the matron was referred to as "a woman of high ability who had done splendid service."

---

A visiting nurse for patients able to pay a moderate fee but unable to afford the whole-time services of a nurse, has been appointed on the staff of the Norwich District Nursing Association. The committee have evidence to show that this meets a real need, and we believe they will find their visiting nurse has come to stay, and will be a precursor of others. It is a practice which is followed in many of the Queen's Homes in other cities, for this kind of nursing seems to succeed better when carried out under an association than as a free lance. The Norwich Association for the first time in its history has a balance on the right side of £248, but has plenty to do with the money. Norwich nurses, by-the-by, are working hard to found a residential club and hostel in connection with the College of Nursing. There are many nurses in the city working on the co-operative system unattached to institutions, and once the club is founded all nurses will be welcomed in it whether College members or not. There is to be a Jumble Sale and White Elephant Stall in aid of this movement on July 2, and Miss V. A. Rutter, 43 Newmarket Road, Norwich, will be glad to hear from any who can help.

---

An arrangement has been concluded between the College of Nursing and the Eagle, Star and British Dominions Insurance Company for providing personal accident and illness policies for nurses. Under Table C. N. 1, all accidents and all illnesses are provided against, but in respect of sickness benefits policyholders are limited to European travel. The yearly premium is £3. Under Table C. N. 2, which is world-wide in all respects as to foreign travel, all accidents but only specified illnesses are provided against. The list of illnesses against which nurses may insure in this latter case is fairly comprehensive, but does not include, to name the most important omissions, tuberculosis or neurasthenia. The premium is £1 5s. The Insurance Company agree to pay the Council of the College an agency fee on all premiums received from members of the College under their Accident and Illness Insurance scheme. The amount, after deduction of expenses, will go to form a fund which shall be used to augment the weekly payments in cases of urgent need, and further assist members in broken health. The advantage of this well-thought-out scheme is that members, while securing a policy framed to cover the special risks of their calling, will be directly benefiting each other. It may be added that premiums gradually decrease through bonus reductions.

---

The St. Pancras Guardians have decided to call their North Infirmary the "Highgate Hospital," and their South Infirmary the "St. Pancras Hospital." It appears that considerable confusion is caused by the fact that there are several infirmaries in Highgate and newly appointed nurses are much puzzled to know which they belong to. In one instance new probationers went to the wrong institution, had supper, passed the night there, and did not discover their mistake till after breakfast next morning. All the same, we are sorry they have dropped the word infirmary, which has many fine associations.

---

A message has been received announcing that the first unit of the Women and Children of Russia Relief Fund, inaugurated by Lady Muriel Paget, is proceeding to the Crimea, where the doctors and nurses will devote themselves to hospital work among the refugees. The stores have safely arrived as well as the personnel for Lady Muriel's mission to Dvinsk in White Russia. Some of the brave women who have gone out to fight famine and disease at fearful odds escaped from Russia at the risk of their lives after terrible hardships and have made the first use of their newly restored health to return to the scene of danger and succour the helpless.

---

Twenty-four nurses have sailed from Liverpool to Constantinople for the Island of Lemnos, where Russian refugees from the Near East are being collected. This is the third hospital unit of the British Committee of the Russian Red Cross Society, and it was inspected by Princess Christian, Chairman of the Committee, before departure. Few people realise the extent of the work carried through for the refugees, for whom bases of relief have been established in Finland, Esthonia, Poland, Serbia, Constantinople and Egypt, besides Lemnos. The demands on the funds increase every month; the refugees are often found to be suffering from dangerous diseases, and are always destitute and half-starved.

